# Applications of Non-destructive Technologies for Agricultural and Food Products Quality Inspection

**DOI:** 10.3390/s19040846

**Published:** 2019-02-18

**Authors:** Hany S. El-Mesery, Hanping Mao, Abd El-Fatah Abomohra

**Affiliations:** 1 School of Agricultural Equipment Engineering, Jiangsu University, Zhenjiang, 212013, China; hanyel_mesery@yahoo.com (H.S.E.-M.); 2 Key Laboratory of Modern Agriculture Equipment and Technology, Jiangsu University, Zhenjiang 212013, China; 3 Department of Crop Handling and Processing, Agricultural Engineering Research Institute, Agricultural Research Center, Dokki, 12618, Giza, Egypt; 4 New Energy Department, School of Energy and Power Engineering, Jiangsu University, Zhenjiang 212013, China; abomohra@yahoo.com (A.E.-F.A.); 5 Botany Department, Faculty of Science, Tanta University, Tanta 31527, Egypt

**Keywords:** agricultural products, non-destructive detection, technologies, food, quality

## Abstract

The quality and safety of food is an increasing concern for worldwide business. Non-destructive methods (NDM), as a means of assessment and instrumentation have created an esteemed value in sciences, especially in food industries. Currently, NDM are useful because they allow the simultaneous measurement of chemical and physical data from food without destruction of the substance. Additionally, NDM can obtain both quantitative and qualitative data at the same time without separate analyses. Recently, many studies on non-destructive detection measurements of agro-food products and final quality assessment of foods were reported. As a general statement, the future of using NDM for assessing the quality of food and agricultural products is bright; and it is possible to come up with interesting findings through development of more efficient and precise imaging systems like the machine vision technique. The present review aims to discuss the application of different non-destructive methods (NDM) for food quality and safety evaluation.

## 1. Introduction

Agricultural and food products with high quality and safety are essential parameters for the consumers, and it is important to introduce strict legislation for food safety and compulsory examination of food products. Therefore, the current food industry has been focused on developing innocuous products that meet the quality requirements demanded by the market, seeking quick and accurate technologies [1]. This resulted in a need to develop harmless food products and constant obligation to the design and application of procedures and methods to precisely control several characteristics in agricultural and food products. However, most of the available investigative methods are slow and destructive to the detected substance. Thus, it is important to develop non-destructive, active, and quick testing techniques to control food quality and safety [2].

Access to good quality and safe agro-food is one of the greatest causes of public anxiety in recent years. Food safety indicates that food includes safe levels of different components, which does not include toxins and contaminants that are injurious to human health. However, food quality includes good appearance such as texture of the food to be favoured by the consumers with high nutritional value [3]. It is essential that all countries guarantee the quality of the imported foodstuff to protect their consumers. In addition to the consumers, more attention should be given to the safety and quality of exported food in the markets worldwide. Therefore, several countries enforce strict control on the standards of food contents which directly affects quality and health. Diseases originating from food is a threat to human health and can bring about decrease in the economic productivity of countries [1]. Currently, non-destructive techniques have been engaged over the past few years to evaluate food quality because they allow the measurement and analysis of different food parameters, reduce wastes and permit repeated measures on the same point over time [4]. On the other hand, application of conventional destructive techniques is recognized to be more labour intensive, time consuming, requires particular material preparation [5]. The visual detecting technologies were examined as possible tools for the above-mentioned objective. The present paper reviewed the recent applications of different non-destructive technologies for evaluation of food and agricultural products quality.

## 2. Safety and Quality of Food

Buzby and Hyman [6] reported that the safety and quality of food are significant parameters which describe parts of foodstuffs. Most fruits and vegetables are healthy and nutritious, which poses no danger of foodborne illness by their consumption. The foods reported to cause illness in some countries were identified as fresh foodstuffs, such as meat [7]. This prompted producers to estimate food quality and classify the dangerous points of food processing to stop any of the reported fresh foodstuff [8]. Thus, quality and safe foodstuff are the main parameters to identify the satisfaction of customers. This is influenced by outside variables such as, appearance (estimate, shape, shading, gleam and consistency), surface, flavour; and different components, which are reviewed by local governments as norms and inward elements [9]. Nonetheless, the description “quality” is extremely broad, suggesting numerous desires that might be different from buyer to another. Quality incorporates properties that impact an item’s value to the customer. Food quality is not exclusively the characteristic of sustenance, but it is also to courses for that characteristics were accomplished. The target elucidation is identified with the product characteristics that portrayed and tested impartially [7,8].

A majority of countries have increased the amount of legislation and requests for food certification. The nature of nourishment is the real standard for the monetary improvement of a nation. Numerous specialists have asserted that security is an essential part of value since an absence of wellbeing can bring about genuine damage and even the demise of the customer. Nevertheless, wellbeing contrasts from numerous other quality properties since it is a quality credit that is not visible. An item can give off an impression of being of top notch (i.e. all around shaded, tempting, flavorful, etc.), however it can be harmful because it may be debased with undetected pathogenic organisms, poisonous chemicals, or physical dangers [10,11].

## 3. Evaluation of Food Quality by Using Non-destructive Methods

The manufacturing of foodstuff is majorly important worldwide. The availability of sufficient quality agricultural producers is a significant problem for the industry, dealers and consumers. Moreover, there is a propensity nowadays for customers becoming needful of additional data regarding the products they purchase due to their increased awareness. The focus on setting up the best needed systems to evaluate the agricultural product quality has been improved. These kinds of interests are related to new technological developments, the growing interest in quality and security of consumer foods, as well as the introduction of more restrictions and standards for nutrition in general [12]. It is difficult to describe the quality of fresh agricultural product because quality could be given different meaning depending on consumer’s inclination [6]. A standardisation to classify the marks of food quality in an item is required for selling the material. The main external and internal parameters of food quality are summarized in Table 1.

Non-destructive methods (NDMs) are a part of high-quality control functions and they support other established techniques. Non-destructive analysis refers to the surface testing of fruits and vegetables without any intrusive technique affecting the food aspect and quality. The non-destructive assessment methods supply data on food characteristics such as structure, mechanical, physical, and chemical properties. The application of non-destructive measurement is the best approach for food processing [1,13]. Antioxidants are materials that can stop the oxidation of other materials and suspensions to protect against cell harm [14]. Specifically, some antioxidants such as anthocyanin, lycopene, and polyphenols extensively occur in several food products, including mulberry, tomato, sweet potato, lychee, and tea. Based on successive projections algorithms for choosing the ideal wavelengths related to anthocyanin content in lychee pericarp, the radial basis function support vector regression and radial basis function neural network models were combined into a single model that demonstrated the best performance in predicting and visualizing anthocyanin content variation in lychee during storage (R^2^ = 0.872) [15,16,17].

**Figure 1:**
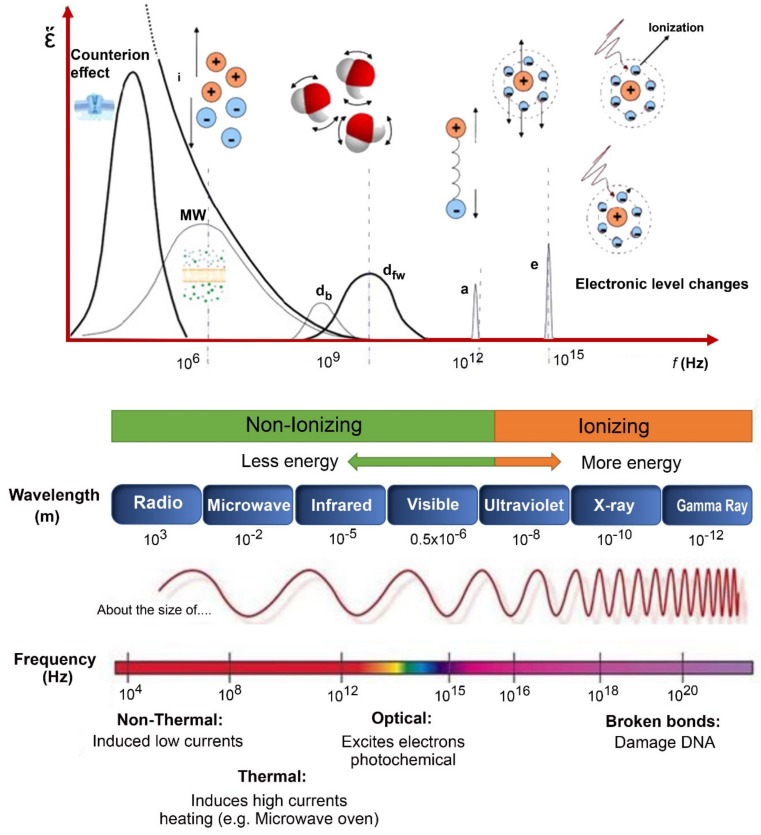
Schematic representation of the electromagnetic spectrum of the different effects that contribute to effective loss factor (modified from [7]).

Table 2 summarizes the most common non-destructive techniques used to test the quality of agricultural products. Regular non-destructive assessment techniques include machine vision, near-infrared spectroscopy, hyperspectral imaging, electronic noses, ultrasound measurement and acoustic emission measurements [3,18,19]. Damez and Clerjon [7] and Wang and Wang [20] have reported that the main components of food are water, carbohydrates, fats, and proteins. Regularly, the processing methods are affected by changes in chemical structure of the agricultural product. The dialectical properties of agricultural products are influenced by cell membranes, the presence of ions, electrical charges on proteins, and pH variations, while cause dielectric spectrum changes as shown in Figure 1.

### 3.1. Machine Vision System

**Figure 2:**
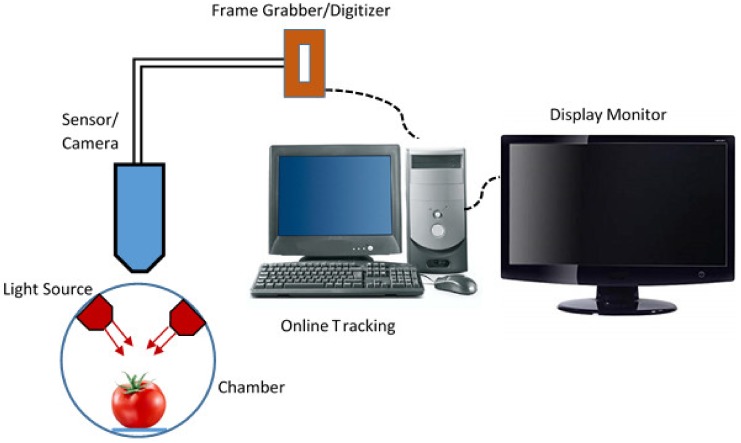
The basic concept and components of a typical machine vision system.

The use of machine vision in food processing has been improved significantly in recent years. Machine vision techniques are automatic, non-destructive, and perfectly suitable for food quality assessment. There are a number of fields in which computers are an intricate part, including terrestrial and aerial mapping of natural resources, crop monitoring, non-destructive evaluation of material quality, etc [21]. In machine vision systems, digital cameras with image analysis systems are used for the automation of visual reviews [22]. The machine vision system usually consists of five basic components: a light, an imaging unit, an image capture board, and the appropriate computer hardware and software [23]. The working principle of machine vision as shown in Figure 2. Machine vision can streamline dreary visual observing processes that take quite a while or require complex mechanisms to be completed. Martynenko [24] confirmed that computer vision techniques showed changes in the density and porosity of ginseng roots during the drying process, consequently providing a strategic alternative to the requirement for checking complex electron optical microscope imaging. However, due to their biological nature the automatic examination of agricultural products has precision issues and problems that are not present in other fields. Although industrial products often have similar colours, shapes and sizes, the same agricultural products may display very different appearances from one item to another. The texture and colour of agricultural products are highly important after harvesting. Besides, the colour of the fruit surface of one piece of fruit may match the colour of an imperfection on the surface of another sample of the same variety [25].

The machine vision technique has been used to examine and estimate foodstuff quality in the food industry. It is affordable, quick, economical, hygienic, and consistent [9,22,26]. Currently, applications of the machine vision technology are commonly used for shape classification, defect detection, and quality assessment. Emadzadeh and Speyer [27] investigated the application of micrometers and image processing systems to compare three Iranian rice varieties, namely Tarom Mahalli, Fajr and Neda. The results showed that the geometric parameters such as length, width, height and the projected area of the three studied varieties decreased while the sphericity increased significantly after removing the outer and the brownish layers. It was found that the values of micrometer data were lower for all the geometric factors.

Machine vision techniques were also applied for quality assessment of cumin and fennel seeds [28]. The method based on discovery minor axis length and zone of the seeds. The classification of good and bad quality was completed on discovery the amount of seeds with basis and foreign elements available in bulk of seeds. The results showed that the quality was inversely proportional to the number of seeds with pedestals (x1) and number of foreign elements (x2) present in the samples. Machine vision systems have been widely used for detection of external pest damages in agricultural products, but because of the challenges involved in the penetration of visible light inside the fruits, it is not effective to detect the internal defects [21]. In that context, an automatic machine vision system was industrialised to detect small insects in raspberry fruit holes by Okamoto et al [29], where the insect infection was difficult to detect even with human eyes. The results indicated that the insects were identified with high success rate, but with some failure cases. In addition, Moradi et al [30] proposed a machine vision system algorithm to control skin colour defects produced by insects based on fuzzy c-means logic with histogram. Application of algorithm showed that the RGB image convert into L × a × b colour space and then active counter model algorithm was used to extract the fruit shape. Finally, the image was segmented to find the defects. The defective pixels were achieved 96% with strong pixels of 91%.

### 3.2. Spectroscopy Detection Techniques

**Figure 3:**
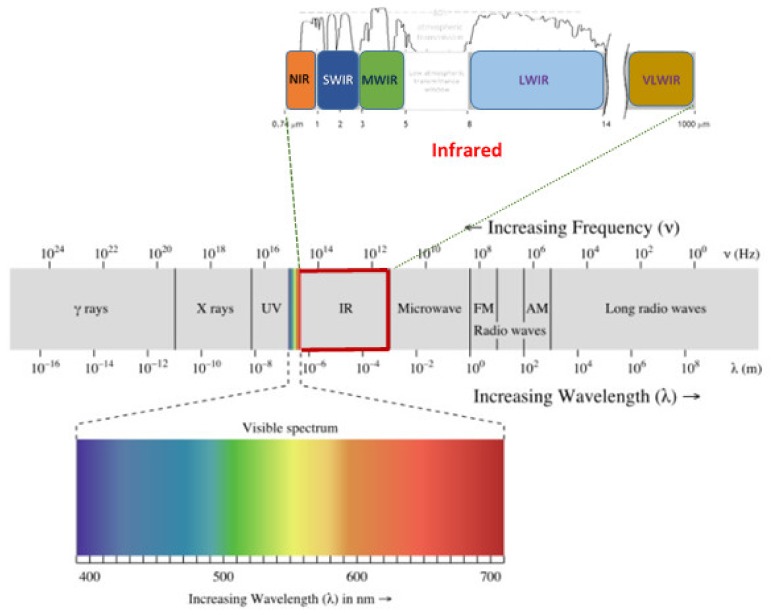
Different spectra of electromagnetic radiation.

**Figure 4:**
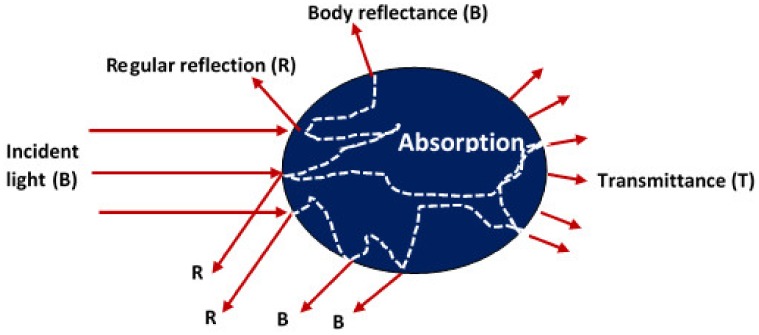
Distribution of incident light on an object showing the reflectance, absorbance and transmittance.

The electromagnetic radiation of photons is divided into radio wave, microwave, infrared, near infrared, visible, ultraviolet, X-ray and gamma-ray ranges [22] as shown in Figure 3. Within the infrared spectrum, the near infrared range of 780 to 1100 nm is known as “Herschel infrared”. Nonetheless, analytical use of spectroscopy in this region has been prevented by the difficulty in understanding the complex absorbance heights and because of its comparatively low energy compared to the visible region. Wei et al. [31] reported that the visual spectrum can be designated according to the reflectance as UV (180–380 nm), visible wavelengths (380–700 nm) and near infrared (NIR, 780–2500 nm). The optical properties are characterized by reflectance, transmittance, absorbance, or scattering. Once the light rays fall on food, about 4% of the incident radiation is reflected by the material surface, while the other part is transmitted through the materials. The absorbed radiation is transformed into other forms of energy instantaneously such as heat, chemical changes or other forms of radiation such as fluorescence and phosphorescence as shown in Figure 4.

The chemical components absorb light energy according to the wavelengths and subsequently the data is collected from spectra tested by using spectrometers. The main absorbers in the NIR wavelengths are carotenoids, anthocyanin, fats, carbohydrates, chlorophylls and proteins. The range of visible wavelengths of food materials from 400 to 750 nm is apparent to humans as colour. The ingredients responsible for the quality features of food such as taste and aroma, in addition to antioxidants, are manufactured in chloroplasts [32]. The non-destructive evaluation of food quality using NIR spectroscopy method has been applied widely for oils, proteins [33,34], dry matter [33,35], firmness [36,37] and total soluble solids [33,36,37,38,39,40]. Application of VIS-NIR spectroscopy for measuring vitamin C content in chillies was also investigated by Wang et al. [41]. Merzlyak et al. [42] studied the reflectance of light on apple fruits by using a spectrometer within a wavelength range of 400–800 nm. Five different apples showed a significant correlation between the reflectance and apple chlorophyll content.

In addition, the application of NIR spectroscopy wavelengths to evaluate the quality parameters of agricultural products have been investigated by several researchers. McGlone et al. [43] reported that using of NIR spectroscopy with NIR spectroscopy region of 750-1100 nm for internal quality evaluation of mandarin fruit, while Bergaz et al. [44] evaluated the NIR spectroscopy for soluble solid content and acidity of mandarin with the range of 350-2500 nm, with correction models to predict SSC being R^2^ = 0.94, root mean square error of prediction (RMSEP) was 0.33 for Brix and R^2^ = 0.80, RMSEP = 0.18% for acidity.

Tian et al. [45] reported that the best wavelength range for quality determination of watermelon is in the region of 300–1000 nm. The wavelength in the range of 400–1700 nm to measure the qualities of intact and sliced melons and watermelon by reflectance mode was reported by Flores et al. [46]. The results showed that the development of model was poor for predictive ability of whole fruit such as cantaloupe melon with standard error of cross validation (SECV) = 1.43 Brix and R^2^ = 0.12; while Galia melon SECV was 0.92 Brix and R^2^ = 0.67, respectively.

Sollid and Solberg [47] studied the application of IR spectroscopy with NIR of 700 to 1100 nm to predict fat content in fresh salmon mince. The results showed high R^2^ of 0.99 attained by applying transmission type. Gowen et al. [48] reported that the spectra of skin and scales could be recorded by means of a fibre optic probe. The results were comparably good with a standard error of prediction of 2.0% and 1.45% for dorsal and ventral lipids, respectively. Currently, NIR spectroscopy is a precise and fast method for the evaluation of fat components in tuna fish, with R^2^ of 0.95 and 0.96 for total fat and free fat, respectively [49]. The possibility of using NIR spectroscopy for measurement of fat content in salmon muscle and the correlation between chemical and NIR analysis R^2^ of 0.94 was reported by Folkestad et al [50]. According to the results, the predicted values of lipid content using IR spectroscopy showed acceptable values in the whole fillet portions, while using the fish mince in the measurement resulted in higher accuracy [51].

The important considerations in applying NIR spectroscopy spectra is the visual path length of fruit. The visual path and optical density of fruit can differ significantly due to variations in the size and thickness (rind and shape) of fruit [52]. Krivoshiev et al. [53] reported that the flesh optical density spectra vary depending on the changes in rind optical density. Chen and Nattuvetty [54] observed that the rind spectrum is always present within the spectral data used for quality evaluation, while the thickness of the optical barrier affects the penetration depth defined. Light depth in apple fruit was studied using NIR spectroscopy by Lammertyn et al. [55]. They concluded that the wavelength penetration varied, being up to 4 mm in the 700–900 nm range and between 2 and 3 mm in the 900–1900 nm range. However, Fraser and Greenhalgh [56] reported that penetration depth of NIR spectroscopy in apple at 700–900 nm more than in the 1400–1600 nm range, mostly because of water absorption. According to the authors, the different results were due to the high water content in fresh fruit, which prevents enough light penetration outside the 200–1200 nm.

### 3.3. Hyperspectral Imaging Technology

**Figure 5:**
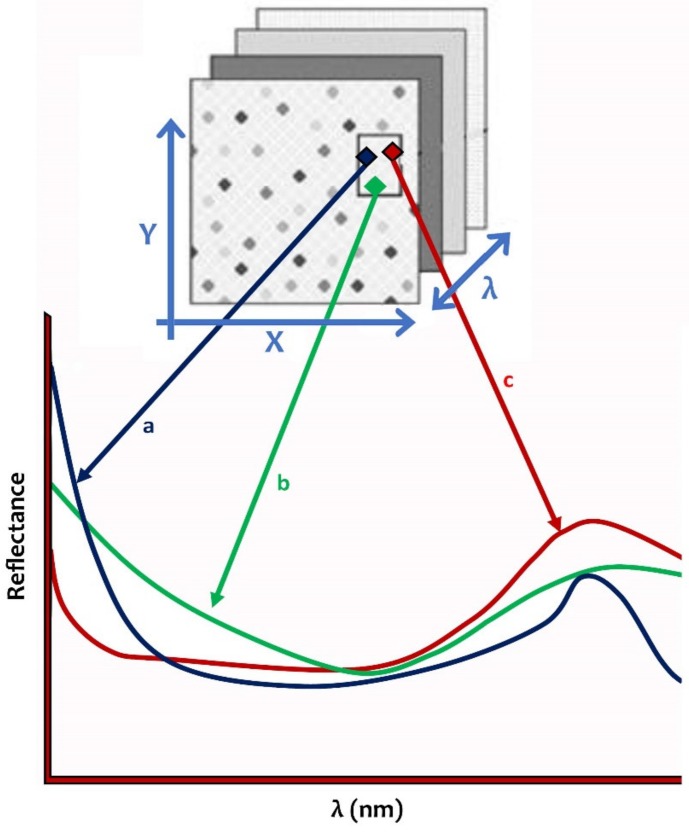
Hypercube representative pixel in a hyperspectrum (modified from [63]).

Hyperspectral imaging methods have been used for non-destructive evaluation of foodstuff quality. Hyperspectral imaging is also named spectroscopic imaging. A hyperspectrum is an influential spectroscopic system for non-destructive analysis that includes the recording of different numbers of images for different spectral groups. The amalgamated images and spectroscopy show concurrently the physical and geometrical properties of the food [57,58]. Through material investigation, exposure to electromagnetic radiation creates spatial maps named hypercubes [59,60], three-dimensional data including two spatial dimensions and a spectral dimension (Figure 5). Each hypercube contains 50–300 of images attained at different wavelengths with a spectral range of 1–10 nm. The fundamental standard which is practical is to combine the spectral data such as (1) the successive acquisition of two dimensional images with different wavelengths, and (2) getting the range of each area of an image in specific spectral region [61,62].

The hyperspectral imaging basic principle is that the food reflects, scatters, absorbs and produces electromagnetic energy in different forms at particular wavelengths because of the differences in food quality. As shown in Figure 5, the reflectance is plotted against the wavelength and the curve is referred to as the spectral signature of that constituent. The changes in the application of the chemical elements of food material have different wavelength reflectance. As shown in Figure 5, the constituents are different in different parts of the reflectance in the spectrum because of the differences in chemical properties

**Figure 6:**
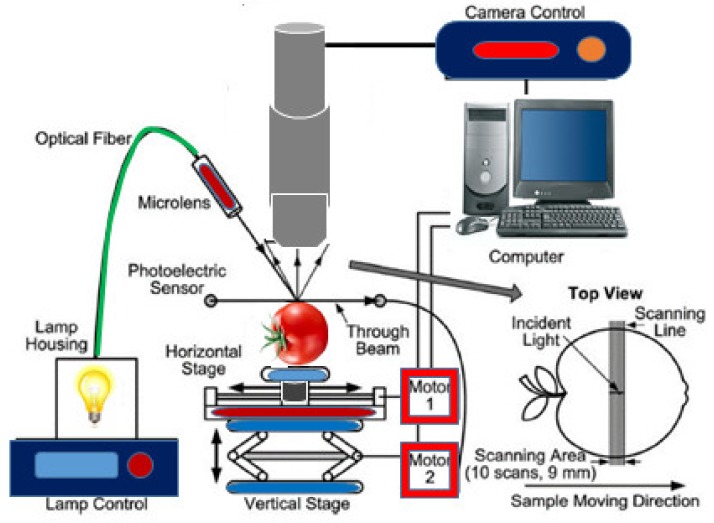
Hyperspectral imaging system for acquiring spatially resolved scattering images from a fruit sample (modified from [65]).

Hyperspectral imaging supplies a large pool of data according to the physical and chemical structure of an imaged material. Hyperspectral imaging as a synchronous source for obtaining spatial images in several spectral groups has been reported by Schaepman [64]. The hyperspectral imaging system contains a hardware and a software part with specific configuration changes created depending on the material and the image acquisition technique used. The hyperspectral imaging system contains a lighting source, a sensor which concurrently acquires spectra, a spectrograph, and a computer to produce the acquired images as shown in Figure 6 [65]. The applications of hyperspectral imaging in food analysis have been investigated in different studies. Hyperspectral imaging offers advantages such as speed, accuracy, and being a non-destructive analysis method that can be used along with the different production ways, concurrent valuation, and real-time information processing of a material’s chemical and physical properties [57,66].

Hyperspectral imaging combined with chemometric analysis is a suitable technique for measurement of lycopene and total polyphenols content. Xiong et al. [67] demonstrated that PLSR was the best model to predict the total polyphenols content in Iron Buddha tea with coefficient of determination for prediction of 0.827. When the BPNN model was used to predict the coefficient of determination it showed high accuracy, with R^2^ up to 0.965, for evaluating total polyphenols content and lycopene contents in whole tomatoes [68].

The possibility of hyperspectral imaging to recognize agricultural and food product contaminants has been studied. The ability of using hyperspectral imaging to estimate forchlorfenuron content in kiwi was reported by Dong et al. [69]. The prediction model based on support vector machines with wavelength range of 928–1658 nm that were selected by successive projections algorithms that showed a top accuracy rate of 97.7%. To differentiate forchlorfenuron-treated kiwi fruits from untreated ones faster, Liu et al. [70] applied five wavelengths (1051, 1230, 1390, 1636, and 1639 nm) that were selected by successive projection algorithms as data for had improved performance compared to the successive projections algorithm model, with a high identification accuracy of 95.0%. Consequently, it is possible to develop an improved forchlorfenuron-treated kiwi identification model based on the hyperspectral imaging method.

A hyperspectral imaging system with spaced wavelengths by first-derivative and a mean centering iteration algorithm was developed and it presented great possibilities for quantitatively discovering different classes of contaminants in foodstuffs like cassava, rye, corn, and common wheat flour [71]. Su et al. [72] investigated the applying hyperspectral imaging to measure two different volatile insecticides on jujube fruits. A regression coefficient method with eight wavelengths (1014, 1038, 1083, 1238, 1288, 1369, 1419, and 1533 nm) as input variables was used to control chlorpyrifos residues and eight different bands (1002, 1032, 1100, 1154, 1184, 1276, 1354, and 1384 nm) were used as input variables to detect imidacloprid residues. Based on the results, the chlorpyrifos, under the same temperature and humidity conditions, was eliminated faster than imidacloprid from jujube surfaces, which increased the final predicted imidacloprid residue R^2^ to 0.806 compared to chlorpyrifos (R^2^ = 0.573).

Early discovery of agricultural crop diseases assists farmers in removing the infected crops before diseases spread and cause more damage [73]. The application of hyperspectral airborne imaging can be a useful and cost-effective way for mapping infected agricultural crops. Use of the 400–1700 nm spectral range reflectance to estimate the presence of the fungus *Helminthosporium maydis* in maize leaves was investigated [74] by taking into account the injuries and colour changes of the maize leaves. The results showed that *H. maydis*-infected maize leaves could be spectrally differentiated using the NIR range 800 to 2600 nm, especially at the early infection level when the symptoms are not yet visible by direct observation.

Hyperspectral imaging in the spectral range of 430 to 900 nm was used to analyse fungal infections of apples [75]. An unequal second difference system applying a chlorophyll absorption band of 685 nm and two bands in the NIR region of 722 and 869 nm provided an excellent detection method for defective/contaminated portions of the apples. Also, the carotenoid absorption band at 450 nm offered a good contrast between healthy apples with respect to samples affected by fungal contamination, and either soil contamination or bruises. The hyperspectral image was exploited to determine firmness, total soluble solids (TSS) and titratable acidity (TA) of mangoes in the spectral region of 450–998 nm with a minimum square regression approach. The performance of the prediction model was examined by analysing R^2^, RMSE and BIAS. A model with high R^2^ values, and with lower RMSE and BIAS results is considered better. The best prediction performance for firmness reached R^2^ of 0.81 and the RMSE (RMSEV) validation of 2.85 N and BIAS of 0.20 N were recorded, similarly for TA, R^2^ of 0.81 and RMSE of the 0.24% and BIAS of 0.00% were estimated. Rungpichayapichet et al. [76] compared the tested parameters and the hyperspectral image showed less predictive performance for TSS, showing R^2^ of 0.5, RMSEV of 2.0% and BIAS of 0.00%, respectively. Teerachaichayut and Ho [77] also used TSS, TA and TSS/TA as a ripeness index for intact limes, with a partial least squares regression model based on the 929–1672 nm spectral range. The accuracy of the models for TSS, TA and TSS/TA displayed coefficients of determination of prediction (R^2^) of 0.838, 0.694 and 0.775, and root mean square errors of prediction (RMSEP) of 0.237%, 0.288% and 0.049%, while the root mean square error of cross validation (RMSECV) were 0.26%, 0.35% and 0.05%, respectively.

**Figure 7:**
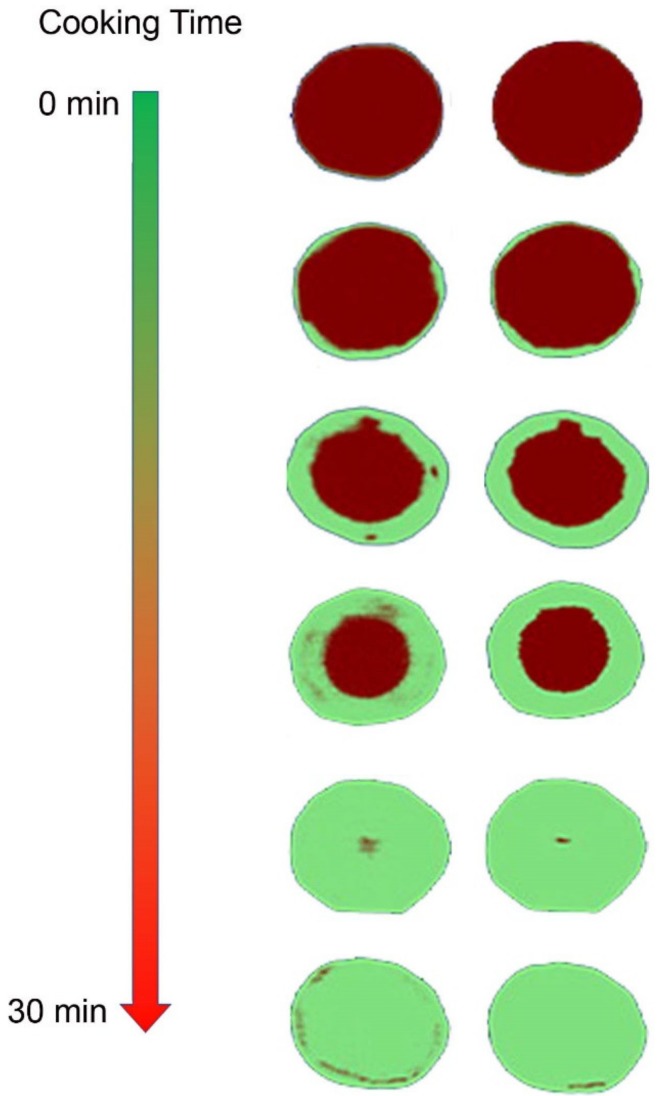
Partial least squares discriminant analysis images of potatoes with different cooking times predicted (modified from [79]).

In addition, applications of hyperspectral imaging techniques to investigate the safety and quality characteristics of foods processed using different methods such as cooking, drying, freezing and storage have been reported. Application of hyperspectral imaging as a non-destructive method for predicting the core temperature (TC) and thermal history (TH) of Japanese seafood using the spectral range of 900–2500 nm was reported by ElMasry and Nakauchi [78]. To build the prediction model, partial least squares regression (PLSR) was used. The results showed high accuracy with R_P_^2^ of 0.86 and 0.83 for TC and TH, respectively. Additionally, the linear discriminant analysis (LDA) algorithm was applied to determine materials where the core temperature reached 65 ^°^C, which represented the cut-off boundary between “cooked” and “uncooked” materials, yielding a classification accuracy of 93.8%. Do Trong et al. [79] studied the hyperspectral imaging HSI method combined with chemometrics to monitor the cooking of potatoes. In the hyperspectral images of the potatoes, the pixels of the raw part, cooked part, and background were assigned values of 2, 1, and 0, respectively, using partial least squares discriminant analysis (PLSDA). The chemical images of cooked potatoes samples which were heated from 0 to 30 min could help monitor the cooking process (Figure 7).

Drying is one of the oldest preservation methods and a predictable food processing technique. Food drying means the removal of water from food products to decrease the water activity, therefore decreasing the rate of microbial spoilage. Drying can be done by many methods such as hot air convection, infrared, microwave and vacuum drying. The drying conditions have significant impact on the physical and chemical quality parameters of food [80]. The most important parameters affected by drying processes are the colour and moisture content of food. Hence, the application of the hyperspectral imaging technique has been used as a fast and non-destructive way to determine the colour and moisture content during the drying processes.

The possibility of using the HSI hyperspectral imaging method for non-destructive measurement of colour components and for the classification of tea leaves during different drying periods was investigated by Xie et al. [81]. Successive projections algorithm (SPA) was applied to select the effective wavelengths, and the least squares-support vector machine (LSSVM) model was established giving encouraging results with correlation coefficients of 0.929, 0.849, and 0.917 for L*, a*, and b*, respectively. Pu and Sun [82] established that HSI hyperspectral imaging in different spectral ranges was a hopeful tool for calculating and predicting the moisture content of mango slices during the drying process. The results showed that the overall optimal HSI model determined had the highest prediction accuracy of R_p_^2^ = 0.972 and RMSEP = 4.611% for the moisture content of mango slices. Also, moisture content of mango at the middle part was lower than those at the corners of the imagining map.

### 3.4. Acoustic Techniques

The acoustic method is an interesting process to evaluate the quality of food and agricultural products. It is quick, economical and non-destructive. In light of such advantages, instrumental acoustic techniques are becoming more common as efficient tools to evaluate the quality of foodstuffs. The acoustic system includes a sound unit, absorption system and techniques to determine the phase oscillation, whereby foods’ quality is measured based upon the sounds created by crushing the food [83]. Utilization of acoustic methods can be quickly classified as involving dissipating or reflecting sound waves which is similar to the dissipation and reflection of light waves. The material receives acoustic pulses from a transducer, then reflects it. Top quality confirmation methods are urgently needed for observing and assessing final food materials to confirm their safety and ensure standard quality [10,11].

**Figure 8:**
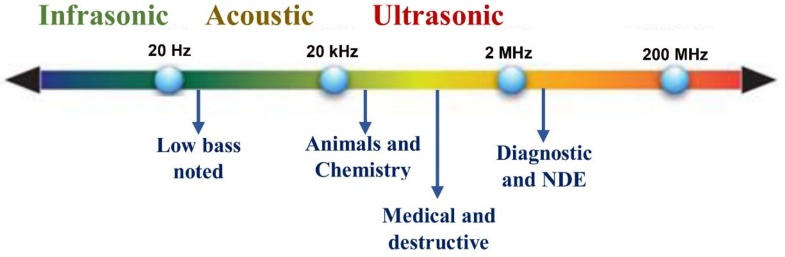
Approximate frequency ranges corresponding to sound, with rough guide of some applications.

The physical reception of sound in any hearing organism is limited to a range of frequencies as shown in Figure 8. Ultrasound is an oscillating sound pressure wave with a frequency greater than the upper limit of the human hearing range. The ultrasound frequency is between 20 kHz and 2 MHz, while the range of human hearing is between 20 Hz to 20 kHz. It is known that soft tissues have a dampening effect on the sound produced when chewing food. Acoustic quality systems are a developing trend for non-destructive quality assessment of agro-foods. Acoustic techniques are based on the response to sound and vibration when the source is softly pinged [84]. The acoustic proceeding is significant for measuring the texture of foodstuffs. The source of acoustic emanation is an unexpected crash and as a result elastic waves proliferate in the foodstuff; however, texture assessment is based upon a number of crashes rather than spread characteristics. In the non-destructive method, the source of acoustic emission is a pendulum [85,86]. Texture properties are a mixture of visual inspection and auditory sensations, where the latter is based on cellular level procedures and the chemical bonds of the cells that influence sounds created by the breakage of cells in crisp foods. The textural characteristics of food gives high acoustic sensations. The force required to chew is responsible for crispness. The waves of sound pressure are created once the cells of materials are cracked [86,87].

The application of a texture analyser coupled with an acoustic device is a novel approach based on the mechanical and acoustic response of the flesh tissue that was investigated by Costa et al. [88]. The results established a good relation between acoustic–mechanical combination in measuring apple crispness and human senses. Taniwaki et al. [89] examined the time course changes in the elasticity index and texture index of two persimmon cultivars during the postharvest period. Zdunek et al. [90] evaluated the texture of apples by developing a new contact acoustic detector and examined models for prediction of the sensory texture of apples. The study showed that the contact acoustic detector was particularly valuable for assessment of sensory crispness, crunchiness and hardness.

**Figure 9:**
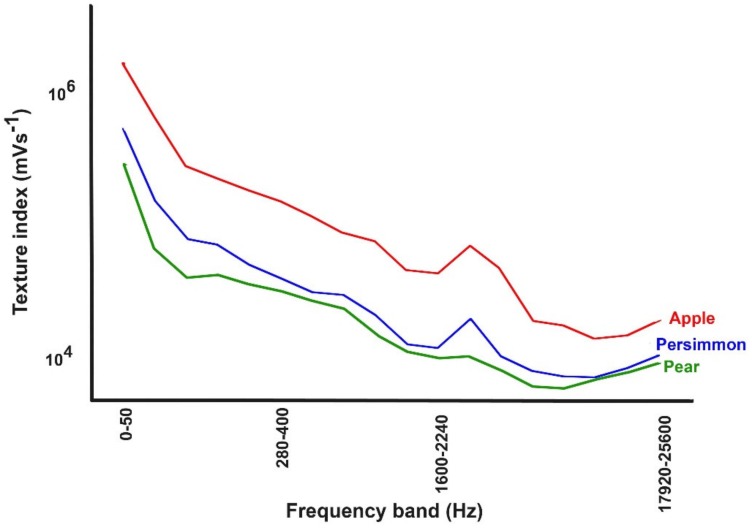
Calculated texture indices for apples, persimmons and pears with texture index versus frequency (modified from [92]).

**Figure 10:**
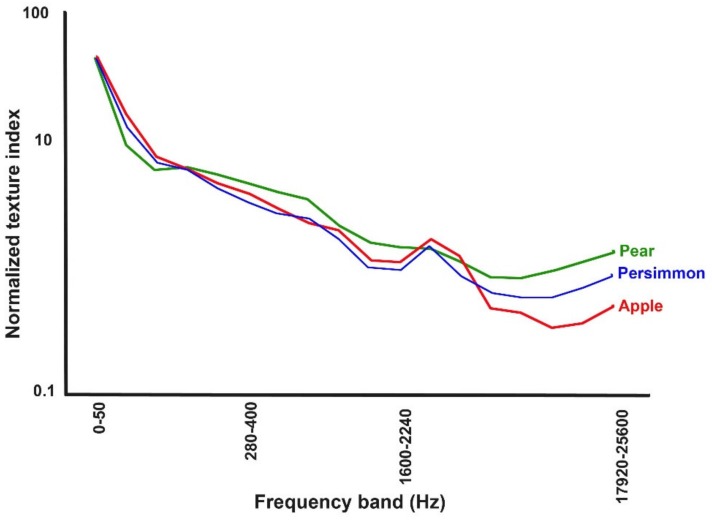
Calculated texture indices for apples, persimmons and pears with texture index versus frequency (modified from [92]).

An application of a piezoelectric sensor in a device to measure and quantify food and fruit texture directly was developed by Iwatani et al [91]. In such a device, a probe is inserted into a food sample (such as apples, persimmons, pears and grapes) where it detects the vibrations caused by the sample’s fracture. Figure 9 and 10 show that the normalized texture indices allow one to compare the textures of different food materials [92]. Although the suggested technique is sufficiently accurate, it is partially destructive and of represents the mechanical properties at a specific measurement point. Diezma-Iglesias et al. [93] reported the influence of storage temperature and time on the firmness process of peach by using non-destructive acoustic. Recently the acoustic impulse response method was applied to evaluate the firmness that is connected to the elastic properties and quality valuation of fruits and vegetables [94].

In general, non-destructive techniques using acoustic methods for sorting, grading and separating agricultural products have been applied gradually and are part of the core research in the field of postharvest engineering [95]. The rapid development of microprocessors, methods of signal analysis and sensors has opened up new possibilities to employ acoustic techniques for this purpose. Profitable interest in sorting food and agricultural products that are more homogenous in quality and consumer favourites has determined a lot of effort for developing this technique. Table 3 summarizes the applications of non-destructive techniques for quality evaluation and safety of agricultural and food products.

## 4. Advantages and Disadvantages of Non-Destructive Techniques

The advantages and disadvantages of non-destructive technique applications in the quality evaluation and safety of agricultural and food products are shown in Table 4.

## 5. Conclusions and Future Perspectives

This paper has been an effort to review the non-destructive detection techniques for evaluation of agricultural product and foodstuff quality. The non-destructive detection for food quality has a characteristic advantage compared to other instrumental analysis and chemical analysis methods and also broad application prospects and development potential. The traditional chemical analysis methods have the following disadvantages: time-consuming, laborious and high cost. In this review, the results show that the utilization of non-destructive techniques provides better advantages for the food industry. The future of using non-destructive technologies for assessing the quality of food and agricultural products will improve the measurement of internal quality characteristics of fruits by developing more efficient and precise imaging systems like machine vision techniques. Although the cost of purchasing such instruments is high at the moment, with collaboration and pooling together research funds from different laboratories, they can be purchased to serve as a central point of investigations for various researchers within a particular geographical region. Another way to solve this is to actively search for ways to develop mini-systems which are equally efficient but with lower costs and easily accessible. In this regard, it is important for researchers from various fields to come on board since the technical challenges associated with this vary from physics to computer science to food and agricultural science. With such combined efforts, it should be possible to produce systems specifically suited for food and agricultural product detection. At the moment, most manufacturers produce machines for different fields of research investigation but with essentially the same system configurations.

## Figures and Tables

**Table 1:** Components of quality factors of fruits and vegetables.

External quality factors	Internal quality factors
*Size*	*Flavour*
Weight, volume, dimension	Sweetness, Sourness, Astringency, Aroma
*Shape*	*Texture*
Diameter/depth ratio	Firmness, Crispness, Juiciness
*Colour*	*Nutrition*
Uniformity intensity	Carbohydrates, Proteins, Vitamins, Functional property
*Defect*	*Defect*
Bruise, stab, spot	Internal cavity, Water core, Frost damage, Rotten

**Table 2:** Most common non-destructive techniques used to test the quality of agricultural products.

Principals	Technique being used	Components
Optics	Image analysis	Size, shape, colour, external defects
	Reflectance, transmittance and absorbance spectroscopy	Colure, chemical constituents, internal defects
	Laser spectroscopy	Firmness, viscoelasticity, defects, shape
Dynamics	Vibrated excitation	Firmness, viscoelasticity, ripeness
	Sonic	Firmness, viscoelasticity, internal cavity density
	Ultrasonic	Internal cavity and structure, firmness, tenderness
	X-ray image and CT	Internal cavity and structure, ripeness
Electro-magnetic	Impedance	Moisture contents, density, sugar content, internal cavity
	MR/MRI	Sugar, oil, and moisture content, internal defects and structure

**Table 3:** Summary of non-destructive applications to evaluate the quality and safety of agricultural and food products.

Products	Technique	Parameters	Spectral range	Reference
Red grape	HSI	Extractable total phenolic content,	900–1700	[96]
Strawberry	HSI	Detection of bruises	650–1000	[97]
Mango	HSI	Skin damage	650–1000	[98]
Peach	HSI	Firmness	500–1000	[99]
Orange	HSI	Soluble solids	700–1100	[100]
Banana	HSI	Soluble solids	400–1000	[101]
Grape	HSI	Total phenols	950–1650	[102]
Tomato	HSI	Firmness and ripeness	500–1000	[65]
Tomato	HSI	Skin damage	1000–1700	[103]
Spinach	HSI	*Escherichia coli* detection	400–1000	[104]
Onion	HSI	Prediction of cooking time	400–1000	[79]
Cucumbers	HSI	Chilling injury	447–951	[105]
Cabbage	HSI	Bacterial contamination	700–1100	[106]
Potato	HSI	Prediction of cooking time	400–1000	[79]
Corn	HSI	Moisture content, Oil content	750–1090	[107]
Wheat	HSI	Detection of insect damage	960–1700	[108]
Wheat	HSI	Identification of classes	960–1700	[61]
Barley	HSI	Analysis of aflatoxin B1	400–2500	[109]
Soy	HSI	Color	400–1000	[110]
Rice	HSI	Growth of *Aspergillus oryzae*	400–1000	[111]
Beef	HSI	Prediction of tenderness	496–1036	[112]
Beef	HSI	Identification and authentication	900–1700	[113]
Beef	HSI	Total viable count of bacteria	400–1000	[114]
Chicken	HSI	Detection of bone in fillets	400–1000	[115]
Chicken	HSI	Detection of diseases	400–900	[116]
Fish	HSI	Oxidation of lipid	400–1000	[117]
Lamb	HSI	Identification and authentication	900–1700	[113]
Cheese	HSI	Prediction of protein; Prediction of fat	960–1662	[118]
Apple	HSI	Detection of bruises	400–1000	[119]
Milk	HSI	Detection of melamine adulteration in milk powder	990–1700	[120]
Milk	HSI	Content of fat	530–900	[121]
Eggs	HSI	Freshness; scattered yolk	380–1010	[122]
Watermelon	NIR	Soluble solid content	700–1100	[123]
Melon	NIR	Soluble solid content	306–1130	[124]
Orange	NIR	Vitamin C	800–2500	[125]
Orange	NIR	Titratable acidity; pH	578–1850	[126]
Passion fruit	NIR	Ascorbic acid; soluble solid content; ethanol	603–1090	[127]
Pomegranate	NIR	pH; soluble solid content	400–1100	[128]
Avocado	NIR	Oil content; moisture content	800–2400	[129]
Pear	NIR	Total soluble solids	990–1700	[34]
Peach	NIR	pH; total soluble solids	800–2400	[35]
cheese	AE	Crispiness		[130]
Biscuit	AE	Crispiness		[131]
Cereal foods	AE	Water content		[132]
Potato chips	AE	Water content		[133]
Apple	AE	Tissue		[134]
Grape	AE	Flesh texture		[91]
Apple	AE	Firmness		[135]
Tomato	AE	Ripening stages		[136]
Mango	AE	Ripening		[137]
Boiled rice	AE	Volume measurement		[138]
Apple	MV	Defect detection		[139]
Oranges	MV	Quality Evaluation		[140]
Strawberries	MV	Sorting		[97]
Papayas	MV	Classification		[141]
Pomegranate	MV	Sorting		[142]
Chilli	MV	Classification		[143]
Wheat	MV	Disease infection		[144]
Corn	MV	Size		[145]
Rice	MV	Grading		[146]
Citrus	MV	Sorting		[147]
Onion	MV	Bacterial infection detection		[148]
Broccoli	MV	Mature		[149]
Bakery	MV	Defects detection		[44]

HSI Hyperspectral Imaging; NIR Spectroscopy; AE Acoustic emission; MV Machine Vision.

**Table 4:** The advantages and disadvantages of non-destructive methods for quality evaluation and safety of agricultural and food products.

Advantages	Disadvantages
**1. NIR Spectroscopy** A non-destructive technique with minimal or no sample preparation required, it allows the determination of chemical and nonchemical (physical) parameters, NIR is rapid and provides real time analytical information from samples, NIR instrumentation is suitable for online use in control processes due to its simplified mechanics and robust components, while fiber optics provide robust sensors for on-line and in-line analysis. **2. Hyperspectral imaging** It provides detailed information about the spectral spatial models for classification and segmentation, Accurate and provides simultaneous analysis of several compounds, Potential to detect diseases and defects within agricultural products.	**1. NIR Spectroscopy** NIR is barely selective, therefore chemometric techniques have to be applied to extract relevant information; accurate and robust models are difficult to obtain as their construction requires large enough number of samples with large variations, NIR requires prior knowledge of the value for a specific parameter which needs to be previously determined using a reference method **2. Hyperspectral imaging** Hyperspectral imaging instrumentation is costly, requires high hardware speed and are complex, Hyperspectral cubes are large and requires significant amount of storage space due to accumulation of vast amounts of multidimensional datasets, Hyperspectral imaging requires chemo-metric techniques to extract relevant information, modelling and data processing is time consuming.
